# Can lumbar hemorrhagic synovial cyst cause acute radicular compression? Case report

**DOI:** 10.1590/S1679-45082014RC3008

**Published:** 2014

**Authors:** Luciana Sátiro Timbó, Laercio Alberto Rosemberg, Reynaldo André Brandt, Ricardo Botticini Peres, Olavo Kyosen Nakamura, Juliana Frota Guimarães

**Affiliations:** 1 Hospital Israelita Albert Einstein, São Paulo, SP, Brazil.

**Keywords:** Spinal diseases, Synovial cyst/surgery, Synovial cyst/complications, Hematoma, Nerve compression syndromes/etiology, Magnetic resonance imaging, Case reports

## Abstract

Lumbar synovial cysts are an uncommon cause of back pain and radiculopathy, usually manifesting with gradual onset of symptoms, secondary to involvement of the spinal canal. Rarely, intracyst hemorrhage occurs, and may acutely present as radicular - or even spinal cord - compression syndrome. Synovial cysts are generally associated with degenerative facets, although the pathogenesis has not been entirely established. We report a case of bleeding complication in a synovial cyst at L2-L3, adjacent to the right interfacet joint, causing acute pain and radiculopathy in a patient on anticoagulation therapy who required surgical resection.

## INTRODUCTION

Lumbar cysts are generally related to degenerative changes in the facet joint , although their pathogenesis is still controversial.^(1-8)^ They may be a cause of back pain and radiculopathy. Although some cases in cervical and thoracic spine have been reported, most cysts are found in the lumbar region, mainly at L4-L5, given the movement of the lumbar spine is greater and more susceptible to instability.^(7,8)^

The hemorrhagic variant is rare. Few cases of acute intracyst hemorrhage were described in the literature.^(1,3-6)^ For example, a total of 254 cases of spinal synovial cysts were reported in the English language literature, but only eight were associated with bleeding.^(9)^ Intracyst hemorrhage is likely to lead to pronounced compression of nerve roots, explaining the acute symptoms in patients.^(4,9)^

We report a case in which hemorrhage of a synovial cyst at L2-L3, adjacent to the right interfacet joint, was responsible for acute back pain and radiculopathy in a patient on anticoagulant treatment, and who required surgical resection.

## CASE REPORT

**Figure 1:**
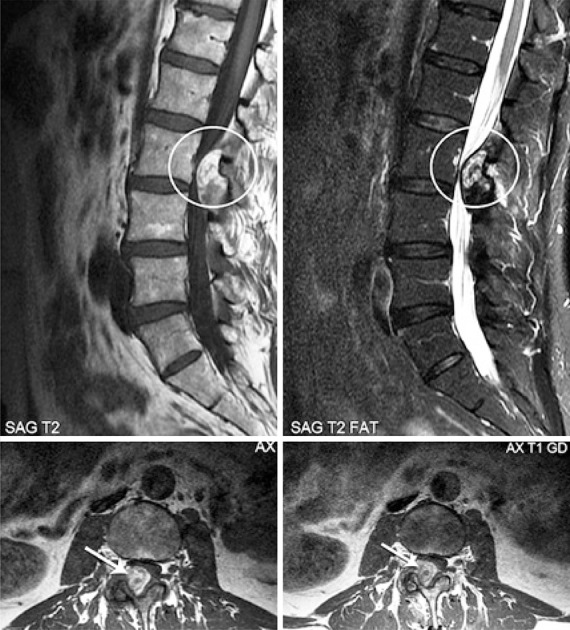
Magnetic resonance imaging showing heterogeneous intraspinal and extradural mass, contiguous to the interapophyseal joint at L2-L3, on the right, with radicular and dural compression, with no signs of fat. Parietal enhancement after contrast

**Figure 2:**
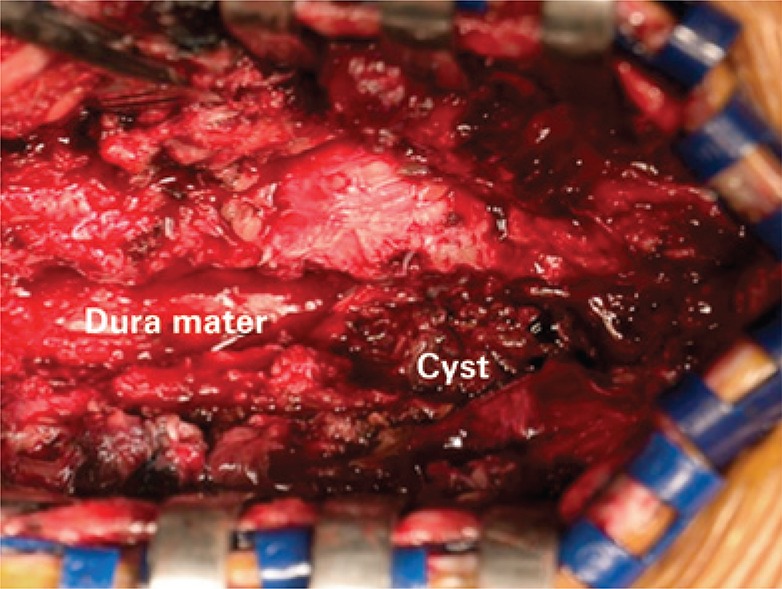
Intraoperative aspect of extradural hemorrhagic cyst at L2-L3, compressing the dura mater, exposed by laminectomy

**Figure 3:**
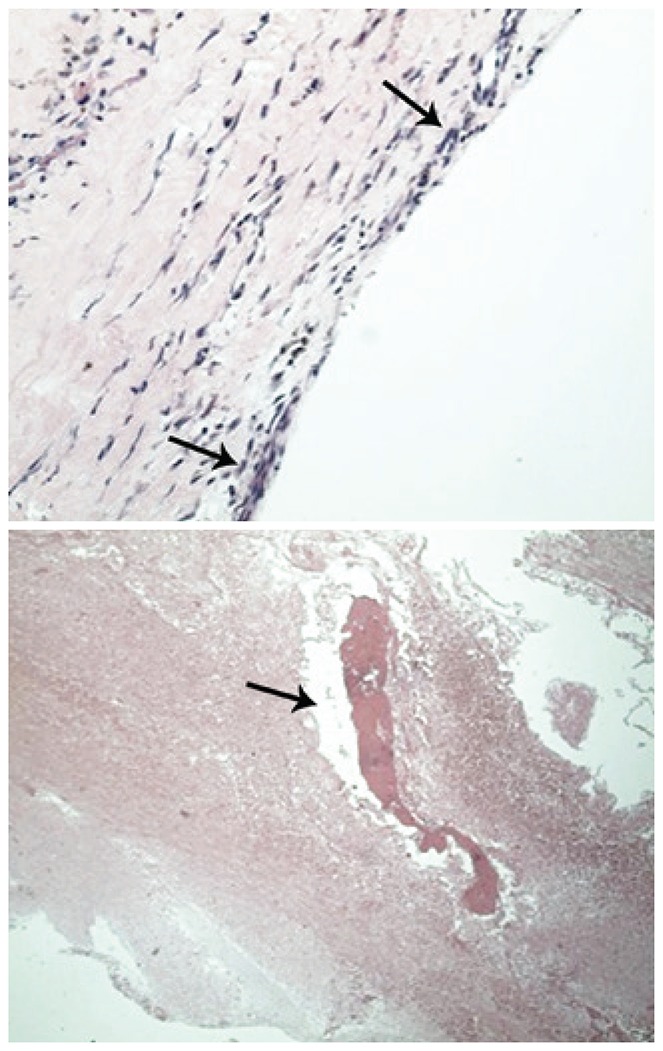
Microscopic view with cyst structure of fibrous walls, lined by flat synovial epithelium without atypia, and with organizing hemorrhagic areas. No signs of malignancy

A 67-year-old male patient with mild and chronic back pain, presented with worsening of pain, with initial irradiation to the right lower limb for 2 weeks, without trauma. The patient had a celiac trunk stent placed 4 years earlier due to an aneurysm, and was on oral anticoagulation (acetylsalicylic acid 81mg every 2 days). The physical examination revealed uncomfortable side movements and rotational movements of the lumbar spine; there was slight hypoesthesia on the dorsal face of both feet and of the first three toes. The magnetic resonance imaging (MRI) of the lumbar spine revealed a large volume intracanal synovial cyst at L2-L3 with hemorrhagic content, causing stenosis of the vertebral canal and compression of the dural sac and of the lateral recess of the right root at L3, in addition to diffuse degenerative spondyloarthropathy (Figure 1). The cyst was not visible on the MRI six months earlier. Surgical intervention was necessary, and a L2-L3-L4 laminectomy and cyst exeresis were performed (Figure 2). The histological study confirmed the diagnosis of synovial cyst with hemorrhage (Figure 3). At the one-month post-operative control, the patient was asymptomatic.

## DISCUSSION

Synovial and ganglion cysts are common lesions that typically occur on limbs. On the vertebral spine, they are known as juxtafacet cysts and are considered rare.^(8)^ They can be defined as a mass of extradural soft tissue along the medial edge of a degenerated facet joint.^(4)^

Synovial cysts are filled with clear or yellowish fluid, have an epithelial lining similar to the synovia, and a demonstrable communication with the joint capsule. If the synovial lining and the joint communication are not evident, the lesion is classified as a ganglion cyst.

These cysts are usually associated with degenerative disease of the spine, but their etiology may be trauma, inflammatory or congenital.^(1,2,7,8)^ Clinical presentation of any juxtafacet cyst depends on the size and location, and on the relation with adjacent structures.^(3)^ There are asymptomatic synovial cysts discovered by chance, as there are also symptomatic ones that cause pain and radiculopathy, generally with chronic development, cauda equina syndrome or, less frequently compressive spinal cord syndrome.^(3,6,7)^

Acute onset of neurological deficits and/or symptoms of pain after hemorrhage in lumbar juxtafacet cysts have been reported.^(1,3-6)^ Exacerbation of pain may be caused by acute hemorrhage inside the cyst, although it has not been clearly determined if the increased pain is due to expansion of a cyst, leading to radicular compression, or due to inflammation resulting from hemorrhage.^(7,8)^ Ramieri et al.^(1)^ mentioned that the expansion of the cyst, after hemorrhage is the cause of symptoms, because this occurs even in rare cases of fast growth of the cyst without bleeding. But, in fact, the cause of the acute onset of symptoms in the sudden intracyst hemorrhage is still under debate.^(1)^

Some factors have been appointed as predisposing to hemorrhage, such as anticoagulation treatment, trauma, disc herniation, vascular anomaly or still neoangiogenesis on the walls of the cyst, secondary to inflammation.^(1,3-6)^ The high vascularization of the cyst in the presence of micro trauma or spinal instability alone are suggested to be sufficient to originate hemorrhage.^(4)^ There have been reports of intracyst bleeding events with no associated trauma or coagulopathy, suggesting that studies should be stimulated, in order to discover new risk factors involved.^(1,3-5)^ In our case, it was not possible to definitively correlate the presence of the hemorrhagic cyst with anticoagulation treatment, because the patient was already on acetylsalicylic acid much before the acute onset, and there are no data that show failure in the patient´s coagulation profile nor in platelet function.

Imaging studies of synovial cysts are useful and should be performed for the differential diagnosis with disc herniation, metastases, meningioma, schwannoma, neurofibromas with cystic degeneration, arachnoid, and perineural, dermoid cysts.^(6-8)^

On T1-weighted MRI sequences, the cysts present as lesions with a low to intermediate signal. At T2, they present high signal content, generally limited by a capsule, which appears as a hypointense line. It should be underscored, however, that, according to the composition of cysts, the intensity of the signal may be heterogeneous, due to the presence of hemorrhage, calcification and gas (vacuum phenomenon). In cysts in which hemorrhage occurs, in the sub-acute phase, metahemoglobin leads to a high signal on all sequences, when compared to non-hemorrhagic cysts.^(7)^ After an intravenous injection of paramagnetic contrast, enhancement of the cyst wall and of its content and, sometimes, enhancement of the adjacent inter-facet joint have been observed.

Treatment of synovial cysts involves both conservative measures, and surgical intervention. The natural history of synovial cysts of the spine is unknown.^(4)^ Reduction and/or spontaneous resolution of cysts may occur with rest and immobilization. Non-hemorrhagic synovial cysts are occasionally treated by percutaneous suction with successful resolution of symptoms. Injection of corticoid in the facet joint may be an option. Percutaneous interventions, including cyst suction, injection and/or rupture, are the most non-surgical treatments studied in the literature. Results vary with success rates between 20 to 75% and, in general, approximately 50% of patients reach significant relief until surgical treatment.^(10)^

Surgical excision with decompression is the definite treatment for lumbar hemorrhagic synovial cysts.^(4)^ Certain cases with acute symptoms require emergency surgery. Even when such surgery is performed fast, some patients still remain with a neurological deficit.^(3)^ However, in general, the immediate diagnosis and appropriate surgical approach may attain satisfactory results.^(4,6) ^Lyons et al.^(2)^ reported the results of 147 patients treated surgically for symptomatic lumbar synovial cysts, and the results at 6 or more months after surgery were from good to excellent in 91%, with most improving motor and sensory functions.

## CONCLUSION

Lumbar synovial cysts are a rare, but possible cause, of acute radicular compression, mainly, when they present hemorrhagic complication. Magnetic resonance imaging is the imaging test of choice to diagnose them and exclude other causes. Although percutaneous intervention may be tried, the results vary much. The immediate acknowledgement and appropriate surgical treatment provide, in general, excellent results.
