# Supplementary material for: Can lumbar hemorrhagic synovial cyst cause acute radicular compression? Case report
Source: Einstein (Sao Paulo). 2014 Oct-Dec;12(4):509–12. doi: 10.1590/S1679-45082014RC3008 (PMC4879922; doi:10.1590/S1679-45082014RC3008)
Supplement: Translation

# Translation: O cisto sinovial hemorrágico lombar pode causar compressão radicular aguda? Relato de caso

**Authors:** Luciana Sátiro Timbó, Laercio Alberto Rosemberg, Reynaldo André Brandt, Ricardo Botticini Peres, Olavo Kyosen Nakamura, Juliana Frota Guimarães

**Affiliations:** 1 Hospital Israelita Albert Einstein, São Paulo, SP, Brasil.

**Keywords:** Doenças da coluna vertebral, Cisto sinovial/cirurgia, Cisto sinovial/complicações, Hematoma, Síndromes de compressão nervosa/etiologia, Imagem por ressonância magnética, Relatos de casos

## Abstract

Cistos sinoviais da coluna lombar são uma causa incomum de dor na coluna e radiculopatia, geralmente com evolução gradual dos sintomas, que são secundários ao comprometimento do canal vertebral. Raramente, há hemorragia intracística, que pode se manifestar de forma aguda com síndrome compressiva radicular ou mesmo medular. Habitualmente, os cistos sinoviais associam-se a doença degenerativa facetária, embora a patogênese não esteja completamente estabelecida. Relatamos aqui um caso em que uma complicação hemorrágica em um cisto sinovial no nível L2-L3, adjacente à interfacetária direita, causou dor lombar e radiculopatia em um paciente em terapia anticoagulante, sendo necessária a ressecção cirúrgica.

## INTRODUÇÃO

Cistos na coluna vertebral geralmente estão relacionados a alterações degenerativas da faceta articular, embora sua patogênese ainda seja discutível.^(1-8)^ Podem ser uma causa de dor lombar e radiculopatia. Embora tenham sido relatados na coluna cervical e torácica, a maioria deles é encontrada na lombar, principalmente em L4-L5, pois a movimentação da coluna lombar é maior e mais suscetível à instabilidade.^(7,8)^

A variante hemorrágica é uma ocorrência rara. Poucos casos de hemorragia aguda intracística têm sido descritos na literatura.^(1,3-6)^ Por exemplo, um total de 254 casos de cistos sinoviais da coluna vertebral foram relatados na literatura inglesa, mas apenas oito foram associados com hemorragia.^(9)^ É provável que o sangramento intracístico conduza à compressão acentuada das raízes dos nervos, justificando a sintomatologia aguda dos pacientes.^(4,9)^

Relatamos um caso no qual a hemorragia em um cisto sinovial no nível L2-L3, adjacente à articulação interfacetária direita, foi responsável pelo quadro agudo de lombalgia e radiculopatia num paciente em terapia anticoagulante, sendo necessária a ressecção cirúrgica.

## RELATO DO CASO

Paciente masculino com 67 anos, com lombalgia leve e crônica, apresentou acentuação do quadro álgico, com início de irradiação para membro inferior direito há 2 semanas, sem trauma. Portador de *stent* no tronco celíaco por aneurisma há 4 anos, em uso de anticoagulação oral (ácido acetilsalicílico 81mg a cada 2 dias). Ao exame físico, observou-se desconforto aos movimentos de lateralização e de rotação da coluna lombar; havia discreta hipoestesia na face dorsal de ambos os pés e dos três primeiros dedos. Ressonância magnética (RM) da coluna lombar evidenciou volumoso cisto sinovial intracanal no nível de L2-L3 com conteúdo hemorrágico, determinando estenose do canal vertebral e compressão do saco dural e da raiz direita de L3 no recesso lateral, além de espondiloartropatia degenerativa difusa (Figura 1). Esse cisto não foi visualizado em RM de seis meses atrás. A conduta cirúrgica foi necessária, sendo realizada laminectomia L2-L3-L4 com exérese do cisto (Figura 2). Estudo histopatológico confirmou tratar-se de cisto sinovial com hemorragia (Figura 3). Já no controle pós-operatório de um mês, o paciente encontrava-se assintomático.

**Figura 1:** Ressonância magnética mostrando massa heterogênea intraespinhal e extradural, contígua à articulação interapofisária de L2-L3 à direita, com compressão radicular e dural, sem sinais de gordura. Realce parietal após contraste

**Figura 2:** Aspecto intraoperatório de cisto hemorrágico extradural em L2-L3, comprimindo a dura-máter, exposta por laminectomia

**Figura 3:** À microscopia, estrutura cística de paredes fibrosas, revestidas por epitélio sinovial plano sem atipias com áreas hemorrágicas em organização. Sem sinais de malignidade

## DISCUSSÃO

Cistos sinoviais e ganglionares são lesões comuns que ocorrem tipicamente nas extremidades. Na coluna vertebral, são conhecidos como justafacetários e considerados raros.^(8)^ Podem ser definidos como uma massa de tecido mole em localização extradural ao longo da borda medial de uma faceta articular degenerada.^(4)^

Cistos sinoviais são preenchidos com líquido claro ou xantocrômico, têm um revestimento epitelial semelhante à sinóvia e uma comunicação demonstrável com a cápsula articular. Caso o revestimento sinovial e a comunicação articular não sejam evidentes, o cisto é classificado como ganglionar.

Esses cistos são habitualmente associados à doença degenerativa da coluna, mas sua etiologia pode ser traumática, inflamatória ou congênita.^(1,2,7,8)^ A apresentação clínica de qualquer cisto justafacetário depende do tamanho, da localização e da relação com estruturas adjacentes.^(3)^ Existem cistos sinoviais assintomáticos descobertos incidentalmente, como também sintomáticos, causadores de dor e radiculopatia, geralmente com evolução crônica, síndrome da cauda equina ou, menos frequentemente, síndrome compressiva da medula espinhal.^(3,6,7)^

Início agudo de défice neurológicos e/ou sintomas dolorosos após hemorragia em cistos justafacetários lombares têm sido reportados.^(1,3-6)^ A exacerbação da dor pode ser causada por hemorragia aguda no interior do cisto, não se determinando claramente se esse aumento da dor é devido à expansão do cisto, levando à compressão radicular, ou à inflamação decorrente da hemorragia.^(7,8)^ Ramieri et al.^(1)^ mencionaram que a expansão do cisto, após a hemorragia, é a causa dos sintomas, porque isso ocorre mesmo em casos raros de rápido crescimento do cisto sem hemorragia. Mas, de fato, a causa do início agudo de sintomas em hemorragia súbita intracística ainda é uma questão de debate.^(1)^

Alguns fatores têm sido apontados como predisponentes à hemorragia, como terapia de anticoagulação, trauma, hérnia discal, anomalia vascular ou ainda neoangiogênese nas paredes do cisto, secundária à inflamação.^(1,3-6)^ Sugere-se que a alta vascularização do cisto na presença de microtraumas ou apenas instabilidade vertebral seriam suficientes para originar uma hemorragia.^(4)^ Há relatos de eventos hemorrágicos intracísticos sem trauma ou coagulopatia associados, insinuando que estudos devem ser motivados, a fim de aventar novos fatores de risco implicados.^(1,3-5)^ No nosso caso, não foi possível correlacionar definitivamente a presença do cisto hemorrágico com a terapia de anticoagulação, pois o paciente já utilizava ácido acetilsalicílico bem antes do quadro agudo, e não há dados que demonstrem descompensação de seu coagulograma e nem função plaquetária.

Os estudos de imagem são úteis no diagnóstico diferencial dos cistos sinoviais, que deve ser feito com: hérnias discais, metástases, meningiomas, schwannomas, neurofibromas com degeneração cística, cistos de aracnoide, perineurais, derSmoides.^(6-8)^

Na RM, em sequencias ponderadas em T1, os cistos apresentam-se como lesões com sinal baixo a intermediário. Em T2, apresentam conteúdo com alto sinal, geralmente delimitado por uma cápsula, que aparece como uma linha hipointensa. Deve-se ressaltar, no entanto, que, conforme a composição dos cistos, a intensidade de sinal pode ser heterogênea, devido à presença de hemorragia, calcificação e gás (fenômeno do vácuo). Nos cistos em que ocorre hemorragia, na fase subaguda, a meta-hemoglobina leva a um alto sinal em todas as sequências, quando comparados a cistos não hemorrágicos.^(7)^ Após a injeção intravenosa do contraste paramagnético, tem sido observado realce da parede do cisto e de seu conteúdo e, eventualmente, realce da articulação interfacetária adjacente.

O tratamento para cistos sinoviais envolve tanto medidas conservadoras, quanto intervenção cirúrgica. A história natural de cistos sinoviais da coluna é desconhecida.^(4)^ A redução e/ou resolução espontânea dos cistos pode ocorrer com repouso e imobilização. Cistos sinoviais não hemorrágicos ocasionalmente são tratados por aspiração percutânea com resolução bem-sucedida dos sintomas. A injeção de corticóide na articulação facetária pode ser uma opção. Intervenções percutâneas, incluindo aspiração do cisto, injeção e/ou ruptura, são os tratamentos não cirúrgicos mais estudados na literatura. Seus resultados são variáveis com taxas de sucesso entre 20 a 75% e, em geral, aproximadamente 50% dos pacientes alcançam um alívio significativo até o tratamento cirúrgico.^(10)^

Excisão cirúrgica com descompressão é o tratamento definitivo para cistos sinoviais hemorrágicos lombares.^(4)^ Certos casos com sintomas agudos requerem cirurgia de emergência. Mesmo quando tal cirurgia é realizada rapidamente, alguns pacientes ainda permanecem com déficit neurológico.^(3) ^No entanto, em geral, o diagnóstico imediato e a abordagem cirúrgica adequada podem produzir bons resultados.^(4,6) ^Lyons et al.^(2)^ relataram os resultados de 147 pacientes tratados cirurgicamente para cistos sinoviais lombares sintomáticos, e os resultados de 6 meses ou mais após a cirurgia foram de bom a excelente em 91%, com a maioria melhorando as funções motora e sensorial.

## CONCLUSÃO

Os cistos sinoviais lombares são uma causa rara, mas possível, de compressão radicular aguda, principalmente, quando apresentam complicação hemorrágica. A ressonância magnética é o exame de imagem de escolha para diagnosticá-los e excluir outras causas. Embora a intervenção percutânea possa ser tentada, os resultados são bastante variáveis. O reconhecimento imediato e o tratamento cirúrgico adequado proporcionam, em geral, um excelente resultado.
